# Time trend analysis of primary liver cancer incidence in Sihui county of Guangdong Province, China (1987–2011)

**DOI:** 10.1186/s12885-016-2817-9

**Published:** 2016-10-12

**Authors:** Wenyi Liu, Qing Liu, Qihong Huang, Yuqiang Lu, Shanghang Xie, Aihua Lin, Sumei Cao

**Affiliations:** 1 Department of Cancer Prevention Research, Sun Yat-sen University Cancer Center, State Key Laboratory of Oncology in South China; Collaborative Innovation Center Medicine, 21 Qingcaigang, Jianshe 6 road, Guangzhou, Guangdong China; 2 School of Public Health, Sun Yat-sen University, 74 Zhongshan 2 road, Guangzhou, China; 3 Sihui Cancer Institute, Sihui, Guangdong China

**Keywords:** Primary liver cancer, Incidence trend, Joinpoint regression analysis, Age-period-cohort model

## Abstract

**Background:**

Southern China is an endemic area for primary liver cancer (PLC), but it is unclear if rates have changed in recent decades. We evaluated PLC incidence and estimated the effects of age, period of diagnosis, and birth cohort in Sihui City, Guangdong Province, China.

**Methods:**

Age-standardized rates (ASRs) of PLC were examined for both males and females from 1987 to 2011. Joinpoint regression analysis was conducted to estimate the annual percent changes in PLC incidence. The age-period-cohort (APC) model was used to investigate the effects of age, diagnosis period, and birth cohort on the relative risk (RR) of PLC.

**Results:**

A total of 2988 PLC cases were identified in this period, with average ASRs of 51.1/100,000 for males and 11.7/100,000 for females. Joinpoint regression analysis revealed increasing PLC incidence throughout the entire period in both males (average annual change of 1.65 %) and females (0.20 %). RRs increased gradually in both sexes from the youngest age group (30–34 years) to the oldest (80–84 years). In males, the RR decreased during diagnosis period from 1987–1991 to 1997–2001 and remained stable thereafter. In females, RRs fluctuated with diagnosis period throughout the entire period. Incidence tended to increase with birth cohort from 1905–1909 to 1975–1979 in both males and females; however, female incidence plateaued in the youngest cohorts born between 1955 and 1974, while incidence in males increased sharply in the cohorts born between 1965 and 1974. According to APC analysis, the full age-period-cohort (APC) model fit the data best, and the period-cohort (PC) model would be enough to explain variability of rates in females.

**Conclusion:**

The PLC incidence rate in males of Sihui City has increased more significantly than female over the last 25 years. Despite the age effect in male, this trend mainly reflects the effects of risk factors that are present in early life (birth cohort) and period change in both genders.

## Background

Primary liver cancer (PLC) is one of the most common cancers worldwide, ranked the fifth most frequent malignancy in males (age-standardized rate (ASR) of 15.3/100,000) and the ninth in females (ASR of 5.4/100,000) [1]. The geographic distribution of PLC is very uneven, with almost 85 % of cases in developing countries and highest incidence rates in Southeast Asia and sub-Saharan Africa [2, 3]. In most populations, the major histological type of PLC is hepatocellular carcinoma (HCC), followed by cholangiocarcinoma (CC) and combined hepatocellular and cholangiocarcinoma (cHCC-CC) [4].

Up to 80 % of PLC worldwide is associated with chronic hepatitis B virus (HBV) and hepatitis C virus (HCV) infections [5]. Infection by HBV is the most common risk factor for PLC in Asia, and prospective cohort studies have found a 5- to 100-fold increase in HCC risk among persons chronically infected with HBV [5, 6]. Exposure to aflatoxin, a mycotoxin from Aspergillus molds, is the second most common cause in most endemic regions of China [7]. In southern China, including Guangdong and Guangxi Provinces, liver and biliary disorders, especially cholangiocarcinoma, are primarily associated with infection by the liver fluke *Clonorchis sinensis* [8, 9]. Other possible environmental and lifestyle risk factors for PLC have also been identified, such as alcohol-related liver diseases, obesity, and smoking [10, 11].

In China, PLC was the third most common cancer in males (268,757 new cases with ASR of 32.2/100,000) and the fifth most common in females (90,083 new cases with ASR of 10.4/100,000) in 2010 [12]. According to the first national death study, the endemic are located in the southern, southeastern, southwestern and northeastern areas of China, such as Guangdong, Jiangsu, Zhejiang, Guangxi and Heilongjiang [13]. Several studies have reported a decreasing trend in some epidemic regions of China, such as Shanghai, Qidong and Fusui [14–16], but changes in PLC incidence have not been examined in southern China. The objective of our study was to describe the changes in PLC incidence during the last 25-year period (1987–2011) in Sihui, Guangdong Province, southern China. These changes may identify modifiable environmental and lifestyle factors for PLC prevention and control.

## Methods

### Study setting

Sihui is a county-level city located in the central part of Guangdong Province. In 2011, the population of Sihui was 418,097 and >75 % lived in rural residences. The major industries include jade processing, smelting, metallurgic casting, and ceramic manufacturing. Climate in Sihui is warm and humid all year around, and the main economic crops are rice and oranges. According to previous reports, the most common malignancy in Sihui is PLC, accounting for 24.56 % of the total [17, 18]. Thus, PLC is an enormous economic and social burden in this region.

### Data sources

Since the establishment of a national three-level cancer network in 1987, a malignant tumor registration report system has been implemented in Sihui [19]. All cancer cases are regularly reported (approximately once a month) by local general practitioners to the central hospital of each town, and then reported to Sihui Cancer Institute once every 3 months. Health specialists assigned by Sihui Cancer Institute collect the reports, record the information onto predesigned cards, and check the quality of the data. To reduce the possibility of missing reports, cancer institute staff also collect the tumor data from hospitals in Sihui once every half year since 1998. For this study, the demographic data (age composition by sex) from 1987 to 2011 were collected from the population information published by the Sihui Statistics Department.

During the 1987–2011 period, 2988 cases of malignant neoplasm of the liver (ICD10 = C22) were identified from Sihui Cancer Registry Institute. The basic registration data, including sex, age, birth date, family history, pathological basis, diagnostic date, disease stage at diagnosis, International Classification of Diseases-10th revision (ICD-10) code, cause of death, registered identification number, and national identification number, were collected from the cancer reporting cards. Among 2988 cases, 675 cases (22.59 %) were from the histologic diagnosis, 79 cases (2.64 %) were from cytological or biochemical or immune deduction, 1674 cases (56.02 %) were from B-Ultrasonograph or other medical imaging, and 373 cases (12.49 %) were from clinical detection. The number of death certification notifications (DCN) cases was 187 (6.26 %), and the ratio of mortality to incidence (M: I) was 94.88 %. This study was approved by the Institutional Research Ethics Committee of Sun Yat-sen University Cancer Center (YB2016-034). In this study, only annual cancer registration report data was used and no information to identify individual subjects was included.

### Statistical methods

In order to estimate age-standardized incidence rates of liver cancer, data stratified by sex were arranged into five shorter intervals (1987–1991, 1992–1996, 1997–2001, 2002–2006 and 2007–2011) for the time period 1987–2011. All age-adjusted incidence rates were calculated using direct standardization with the Segi’s World Standard Population (1960) [20].

Joinpoint regression was used to estimate annual percent changes (EAPCs). Significant joinpoints were identified by permutation tests using 95 % of the asymptote [21], starting with no joinpoints and adding up to three to the model [22].

An age-period-cohort (APC) model was used to estimate the incidence of PLC. Regression analysis considered the effects of three factors on incidence for each gender: age, period, and cohort [23]. The model assumed that the number of liver cancer cases follows a Poisson distribution and that incidence rates are a multiplicative function of the included model parameters, making the logarithm of the rates an additive function of the parameters [24, 25]. Relative risks (RRs) were calculated to show the effects of age, diagnosis period, and birth cohort in the APC models. All analyses were restricted to the 30–84 years age range and data were categorized into 11 age groups from 30–34 years to 80–84 years when applied to the APC model, together with 5 diagnosis period groups (1987–1991 to 2007–2011) and 15 birth cohort groups (1905–1909 to 1975–1979) (Table 4 in Appendix). To make this model more stable, we chose the 55–59 year age group, the 1997–2001 diagnosis period group, and the 1940–1944 birth cohort group as references instead of the first or the last group [26]. Goodness of fit of the models was assessed both by deviance and Akaike’s information criterion (AIC). Ratios of deviance to the degrees of freedom were closer to 1 and lower AIC values indicated a better fit [27, 24, 25]. All analyses were performed using SAS 9.3 (Version 9.3, SAS Institute Inc., NC, USA).

## Results

### PLC incidence in Sihui

**Fig. 1:**
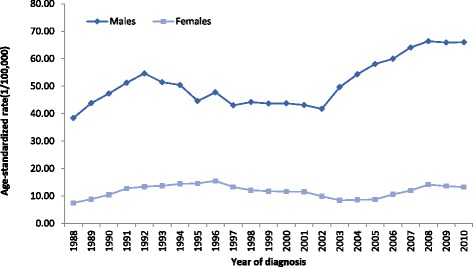
Age-standardized rates of primary liver cancer incidence by sex using the moving average in three-year intervals in Sihui, China, 1987–2011. The overall age-adjusted rate among males and females was increased in this period, from 32.49/100.000 to 69.54/100,000 in male and 7.03/100,000 to 13.43/100,000 in female (Fig. 1)

Over the 25-year study period from 1987 to 2011, 2988 PLC cases were identified in Sihui. Of these, 2380 (79.7 %) were diagnosed in men and 608 (20.3 %) in women. The crude incidences were 33.74/100,000 for the total population, 47.01/100,000 for males, and 12.64/100,000 for females between 1987 and 2011. The corresponding ASRs were 30.83/100,000 for the total population, 51.14/100,000 for males, and 11.69/ 100,000 for females. The overall age-adjusted rates among males and females increased in this period, from 32.49/100.000 to 69.54/100,000 in male and 7.03/100,000 to 13.43/100,000 in female (Table 1, Fig. 1).

**Table 1:** Age-standardized rates of primary liver cancer incidence in Sihui City, Guangdong Province, China from 1987 to 2011

Year	Males			Females		
	Cases	Crude incidence (/100,000)	ASR (/100,000)	Cases	Crude incidence (/100,000)	ASR (/100,000)
1987	50	29.27	32.49	13	7.66	7.03
1988	61	35.30	39.35	12	7.06	6.56
1989	68	39.32	43.50	17	9.79	8.93
1990	80	44.69	48.72	19	10.87	11.14
1991	82	46.07	49.74	23	13.03	11.38
1992	96	52.70	55.36	31	17.46	15.84
1993	102	55.06	58.87	24	13.40	13.09
1994	72	38.19	40.19	24	13.31	12.45
1995	97	50.88	52.39	35	19.22	17.76
1996	70	36.17	41.28	23	12.53	13.62
1997	81	41.29	49.69	29	15.66	15.18
1998	68	33.85	38.25	22	11.67	11.12
1999	78	38.69	44.73	20	10.50	10.16
2000	93	45.92	48.15	31	16.22	14.01
2001	76	37.29	38.42	22	11.44	10.72
2002	84	41.09	42.85	20	10.34	9.86
2003	92	44.72	43.94	21	10.82	9.22
2004	121	58.29	62.15	15	7.73	6.40
2005	117	55.57	56.88	26	13.31	10.30
2006	114	53.82	55.28	21	10.63	9.54
2007	140	66.11	67.72	29	14.67	12.04
2008	140	66.38	69.36	33	16.50	14.52
2009	126	59.62	62.12	39	19.31	16.08
2010	133	62.76	66.30	25	12.30	10.28
2011	139	65.08	69.54	34	16.62	13.43
total	2380	47.01	51.14	608	12.64	11.69

*ASR* Age-standardized rates

### Joinpoint regression analysis of PLC incidence

**Fig. 2:**
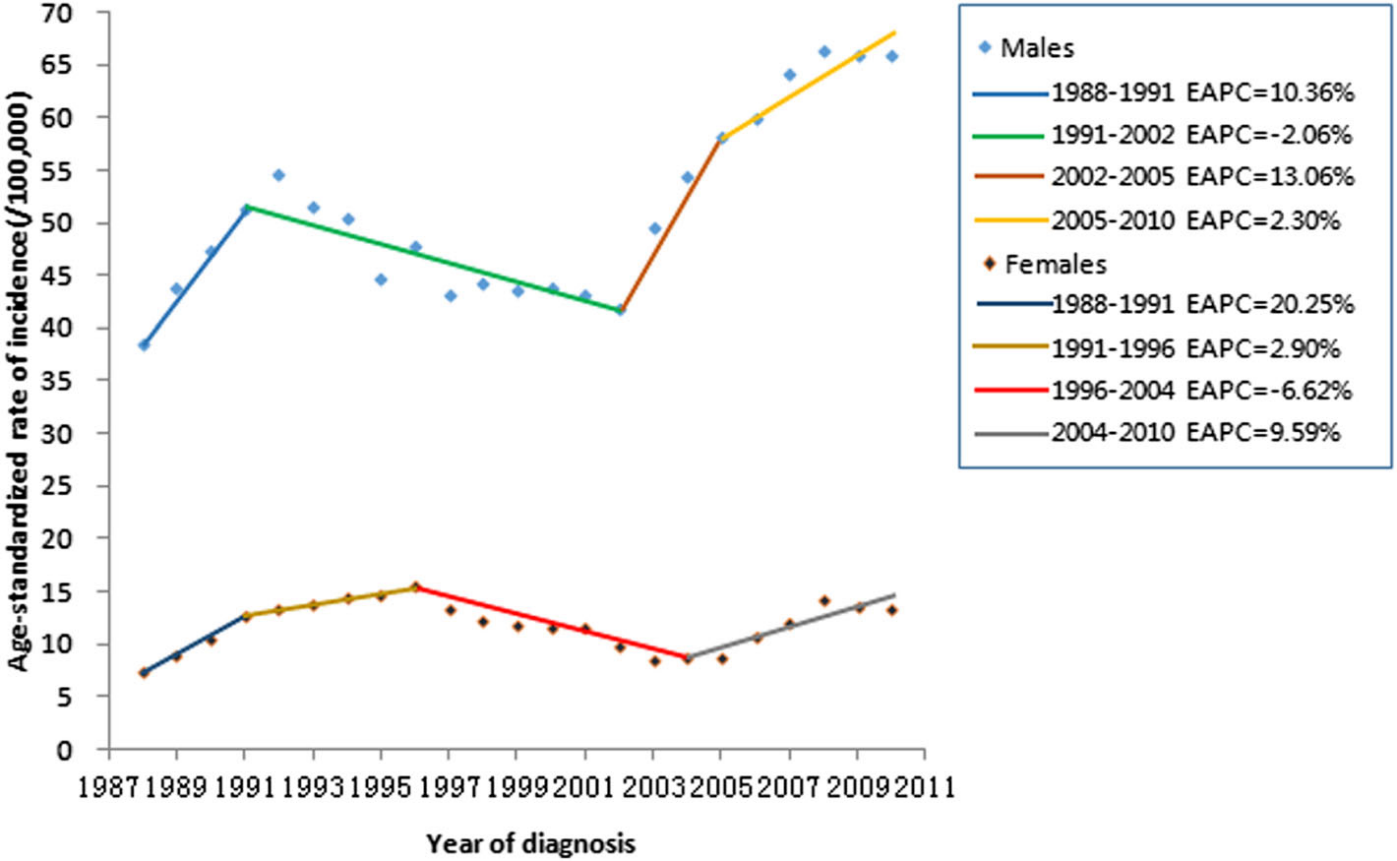
Trends in world age-standardized rates (ASR-W) of PLC incidence for both sexes in the period of 1987–2010 in Sihui, China, with joinpoint regression. During the 25-year period, three-joinpoint model were obtained as the best model for both sexes and the incidence of PLC fluctuated with increasing trend at an average annual percent change of 1.65 % for males and 0.20 % for females (Fig. 2)

During the 25-year period, three-joinpoint model was obtained as the best model for both sexes, and the incidence of PLC fluctuated with increasing trend at an average annual percent change of 1.65 % for males and 0.20 % for females (Fig. 2).

### Age-period-cohort effects on PLC incidence

**Fig. 3:**
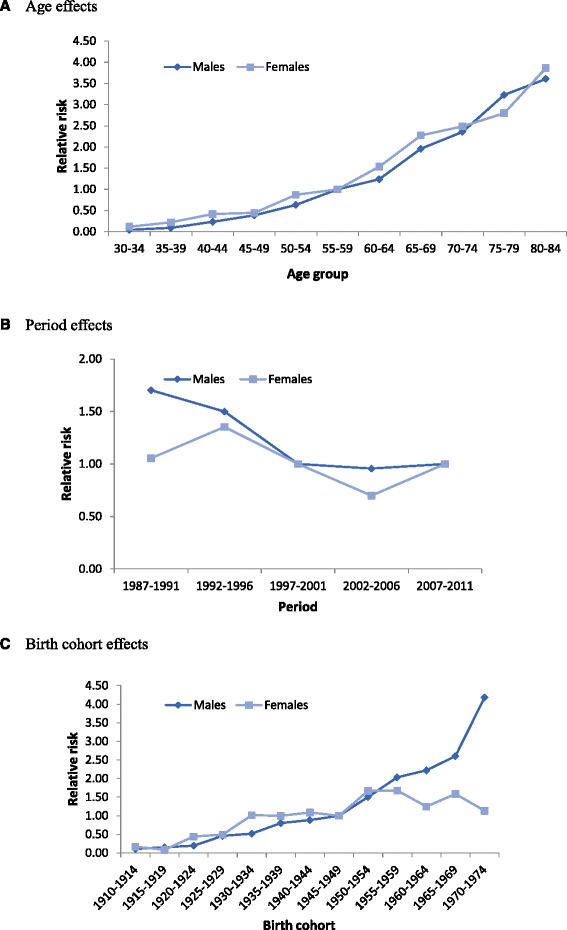
Effect of age, period, and birth cohort on the relative risk of primary liver cancer incidence with APC regression analysis in Sihui county by sex. **a** Age effects; **b**, Period effects; **c**, Birth cohort effects. The age effects for both sexes showed that the risk of PLC increased with age. Compared with the age group of 55–59 years, the RR of PLC was up to 3.61(95 % CI:2.20–5.96) for males and 3,86 (95 % CI:1.78–8.36) for females in the oldest age group (80–84 years) (Fig. 3a). Period effects for males depicted that the RRs decreased from 1987–1991 to 1997–2001, and then plateaued afterwards; for females, RR fluctuated through the entire period. The RRs increased from period1987-1991 to 1992–1996, decreased consistently from 1992–1996 to 2002–2006, and then increased to the staring risk (Fig. 3b). By using the 1940–1944 birth cohort as the reference group, an obvious increased incidence trend in both males and females were obtained; however, females had a plateau in the youngest cohorts who were born during 1955–1974 and males had a sharp increasing trend in the cohorts who were born during 1965–1974 (Fig. 3c)

In males, the full APC model fit the data best with the Deviance =42.96, AIC = 10352. Among females, a period-cohort model was sufficient to explain variability of the PLC incidence, since a non-significant (ΔDeviance) between the age-period-cohort (Deviance = 28.95) and the period-cohort (Deviance =42.19; *p* = 0.152) models was found. The Δdeviance of corresponding models of age-period (Δdeviance = 47.04) was higher than period-cohort (Δdeviance = 13.24) indicated that the birth cohort effect was more important than the age effect in female (Table 2). The component effects in the final APC model are shown in Table 3. The age effects for both sexes showed that the risk of PLC had consistent increasing trends with age. Compared with the age group of 55–59 years, the RR of PLC was up to 3.61(95 % CI:2.20–5.96) for males and 3.86 (95 % CI:1.78–8.36) for females in the oldest age group (80–84 years) (Fig. 3a). Period effects for males depicted that the RRs decreased from 1987–1991 to 1997–2001, and then plateaued afterwards; for females, RRs fluctuated through the entire period (Fig. 3b). By using the 1940–1944 birth cohort as the reference group, an obvious increased incidence trend in both males and females were obtained; however, females had a plateau in the youngest cohorts who were born during 1955–1974 and males had a sharp increasing trend in the cohorts who were born during 1965–1974 (Fig. 3c).

**Table 2:** Age-period-cohort models for PLC incidence rates in Sihui, China

	DF	Deviance	Deviance/DF	ΔDeviance	ΔDF	*P*	AIC
Male							
Age	44	157.34	3.58	114.38	17	<0.001	10432
Period	50	1103.36	22.07	1060.04	23	<0.001	11366
Cohort	40	507.06	12.68	464.04	13	<0.001	10790
Age-period	40	87.76	2.19	44.8	13	<0.001	10370
Age-cohort	30	69.51	2.31	26.55	3	<0.001	10372
Period-cohort	36	140.28	3.9	97.32	9	<0.001	10431
Age-period-cohort	27	42.96	1.59	Reference	Reference	Reference	10352
Female							
Age	44	98.48	2.24	69.53	17	<0.001	11483
Period	50	407.97	8.16	379.02	23	<0.001	11780
Cohort	40	113.21	2.83	84.26	13	<0.001	11505
Age-period	40	75.99	1.9	47.04	13	<0.001	11468
Age-cohort	30	47.2	1.57	18.25	3	<0.001	11459
Period-cohort	36	42.19	1.17	13.24	9	0.152	11443
Age-period-cohort	27	28.95	1.07	Reference	Reference	Reference	11447

*DF* Degree of freedom, *ΔDeviance* increase in deviance from the APC model, *ΔDF* increase in DF from the APC model, *AIC* Akaike’s information criterion

**Table 3:** Relative risk (RR) and 95 % confidence interval (CI) of PLC incidence for both sexes by age-period-cohort model in Sihui, China

Parameter	Subgroup	Males		Females	
		RR	95 % CI	RR	95 % CI
Age (years)	30–34	0.04	0.03–0.07	0.12	0.05–0.28
	35–39	0.09	0.07–0.13	0.22	0.11–0.44
	40–44	0.23	0.18–0.30	0.42	0.24–0.72
	45–49	0.39	0.31–0.48	0.45	0.28–0.73
	50–54	0.63	0.53–0.75	0.87	0.59–1.28
	55–59	Reference		Reference	
	60–64	1.24	1.03–1.49	1.54	1.06–2.21
	65–69	1.96	1.57–2.45	2.27	1.48–3.49
	70–74	2.36	1.78–3.12	2.49	1.48–4.18
	75–79	3.23	2.26–4.61	2.80	1.49–5.26
	80–84	3.61	2.20–5.96	3.86	1.78–8.36
Period	1987–1991	1.70	1.33–2.18	1.05	0.66–1.67
	1992–1996	1.50	1.25–1.80	1.35	0.97–1.89
	1997–2001	Reference		Reference	
	2002–2006	0.96	0.86–1.07	0.70	0.55–0.88
	2007–2011	1.00	0.91–1.25	1.00	0.61–1.52
Cohort	1910–1914	0.11	0.04–0.29	0.17	0.04–0.68
	1915–1919	0.15	0.08–0.34	0.08	0.02–0.34
	1920–1924	0.20	0.12–0.32	0.44	0.20–0.96
	1925–1929	0.46	0.32–0.65	0.49	0.26–0.93
	1930–1934	0.52	0.39–0.69	1.02	0.62–1.67
	1935–1939	0.80	0.64–1.00	1.00	0.65–1.52
	1940–1944	0.88	0.73–1.06	1.09	0.75–1.58
	1945–1949	Reference		Reference	
	1950–1954	1.51	1.24–1.82	1.67	1.12–2.50
	1955–1959	2.03	1.63–2.54	1.68	1.04–2.70
	1960–1964	2.22	1.70–2.90	1.24	0.69–2.23
	1965–1969	2.60	1.88–3.61	1.59	0.78–3.21
	1970–1974	4.18	2.84–6.15	1.13	0.47–2.71

## Discussion

This study reveals that the incidence rate of PLC in Sihui (30.8/100,000) was substantially higher than the national level (10.1/100,000) during the period 1987–2011 [1]. Moreover, the average ASR in this region exhibited an increasing trend over this period, in contrast to three other endemic regions of China, Shanghai [14], Qidong [15], and Fusui [16], where incidence rates are stable or declining. The APC analysis revealed that the increasing trend of PLC incidence in Sihui is primarily due to the effects of period changes and a spike in the younger cohorts, particularly males born between 1965 and 1974. Such a pattern may indicate the impact of lifestyle and environmental changes on the risk of PLC in rural areas of southern China.

Reasons for the high incidence of PLC in Sihui are not completely clear but may be related to higher HBV infection rates and other possible infection risk factors compared to other regions of China. A survey conducted between 1992 and 1995 reported the highest HBV infection rate in Guangdong compared to the rest of China, with HBs-Ag positive rate of 17.85 % in the general population and 19.86 % in children aged <15 years. Among children aged <15, age-specific prevalence rates of HBs-Ag was 23.68, 14.63, 17.5319.6118.71, and 23.84 % in the 6 age groups (1-, 2–3, 4–5, 6–7, 8–9, 10–14 years), respectively [28–30]. Hence, reducing HBV infection is critical for controlling PLC in southern China. Hepatitis B vaccination of neonates and infants is the most effective way of eliciting protective antibodies to HBV surface antigen (anti-HBs) and in reducing the prevalence of HBs-Ag among children [31, 32]. Since 2002, subsidy for HBV vaccination has been provided for all infants and has thereafter largely reduced HBV infection rate among Chinese children [31, 32]. However, HBV infection remains highly prevalent in the population born before the vaccination program and the incidence of PLC has remained high in adults. Since hepatitis B vaccine is proven to prevent HBV infection among children and teenagers, it may also prove beneficial to young adults in preventing HBV induced PLC [31].

*Clonorchis sinensis* (*C. sinensis*) infection is another possible contributor to the high incidence of PLC in Sihui. Clonorchiosis is also common in Guangdong, with heavily endemic areas located along the Pearl River Delta [33]. Raw freshwater fish is a staple of the Cantonese diet in this region, and these fish act as the second intermediate host in the lifecycle of *C. sinensis*. Research showed that *Clonorchis sinensis* infection is strongly related to liver and biliary disorders, especially cholangiocarcinoma [9]. However, we were unable to differentiate between types of PLC because the proportion of histologically diagnosed PLC was small (9.14 %) and only 2.5 % of the histologically diagnosed PLC cases can be classified as a specific pathological subtype. Given the symptoms of PLC are insidious at onset, progress quickly and with high mortality, the disease is usually diagnosed when the tumor is large and incurable. Therefore, patients often refuse the pathological biopsy diagnosis and treatment. Increasing the early detection rate and treatment effect might improve the pathologic diagnostic rate of PLC in the future. Another limitation is we cannot analyze the effect of risk factors clearly for PLC cases due to the lack of environmental risk factors exposure information.

The APC analysis suggests that population aging is one of the effectors of the increasing PLC incidence in both sexes. Indeed, PLC incidence increased progressively with age, peaking in the 80–84 year age group. With economic development and introduction of modern medical technology, the proportion of elderly in the general population has increased dramatically. In 2010, the average life expectancy in Sihui (76.6 years) was longer than the national by 3.1 years [34]. Age increases PLC risk due to the long incubation process (about 50 years) from HBV infection to PLC [35, 36], possibly conferring higher PLC risk for the given infection rate.

Variations in incidence over long periods often reflect the impacts of new diagnostic techniques, improved medical interventions, and greater accuracy in determining the causes of morbidity and mortality. According to the APC model, PLC incidence rates were highest for both sexes in the first diagnostic period included (1987–1991). In females, this period was followed by a general decreasing trend, while in males the rate trended downward only until 1997–2001 and has since remained stable. The peak detection of PLC in the 1987–1991 period may be due to the widespread introduction of ultrasound and AFP measurement [10]. In the 1980s, ultrasound examination together with AFP testing has been gradually popularized as a PLC screening method in the endemic areas in China, which led to a large number of asymptomatic cases of PLC found and more frequent neoplasms identified from cirrhotic patients [37, 38].

A birth cohort effect usually reflects early life exposure to specific risk factors that were absent in other periods [39]. In our study, birth cohort-stratified incidences in males and females showed two trends. Before the 1947–1951 cohort, a slow progressive increase in PLC incidence was observed in both males and females. However, there was a sharp acceleration from the 1952–1956 to the 1968–1972 cohort in younger males, whereas the equivalent female cohorts showed only a narrow fluctuation. We suggest that the combination of high HBV and *C. sinensis* infection rates, exposure to cigarette smoking and alcohol drinking in the younger male generation may be the main reasons underlying this birth cohort effect in males of Sihui. It has been reported that males had higher prevalence rate than females for HBs-Ag (23.56 *vs* 15.55 %, *p* < 0.05) [29] and for *C sinesis* (18.92 *vs* 13.89 %, *p* < 0.05) [33]. Several studies conducted in 1980s-1990s reported that the prevalence rates of cigarette smoking were 15 times higher (39.04 *vs.*2.47 %) and alcohol drinking 21 times higher (38.24 *vs* 1.77 %) in the male younger cohorts than females [40, 41]. These differences might result in a more rapid increase in PLC incidence in men than women. More detailed information on the risk factors for PLC should be gathered from southern China.

## Conclusions

A significant increase in the incidence rate of primary liver cancer was observed in Sihui from 1987 to 2011 for both sexes. Estimates from cohort effect showed that the increasing trend in male is likely to continue over the next decade in male, while female tended to leveled off since 1950. Although the reasons for increasing PLC incidence in Sihui are not fully clear, higher prevalences of HBV and *C. sinensis* infection are likely contributors. Further research exploring the prevalence of possible risk factors associated with PLC is needed to formulate a comprehensive PLC prevention and control strategy in this region of southern China.

## Appendix

**Table 4:** Incidence rates of PLC in different age groups, period groups and birth cohort groups for both sexes in Sihui, China

Variable	Subgroup	Males		Female	
		Cases	Incidence rate (/100,000)	Cases	Incidence rate (/100,000)
Age (Years)	30–34	95	21.87	15	3.67
	35–39	166	41.62	29	7.80
	40–44	252	85.88	45	16.60
	45–49	279	106.90	42	17.63
	50–54	303	140.92	62	31.02
	55–59	311	180.80	60	34.45
	60–64	279	169.30	81	49.21
	65–69	267	208.64	90	57.91
	70–74	181	194.75	71	53.83
	75–79	116	191.44	45	44.89
	80–84	44	139.91	31	40.90
Period	1987–1991	323	34.02	78	8.43
	1992–1996	422	41.82	129	13.49
	1997–2001	379	37.71	379	40.00
	2002–2006	506	48.65	96	9.85
	2007–2011	659	62.20	152	15.09
Cohort	1910–1914	19	103.88	3	8.89
	1915–1919	36	105.69	22	39.58
	1920–1924	116	195.60	32	37.57
	1925–1929	155	172.57	85	67.98
	1930–1934	253	207.60	79	51.63
	1935–1939	268	167.97	77	58.00
	1940–1944	236	138.97	54	32.52
	1945–1949	274	137.20	65	36.82
	1950–1954	207	79.51	57	24.06
	1955–1959	232	74.73	37	12.62
	1960–1964	167	60.89	31	11.87
	1965–1969	141	58.27	15	6.56
	1970–1974	74	37.26	7	3.79

## Abbreviations

AFP: α-fetoprotein
AIC: Akaike’s information criterion
anti-HBs: Antibodies to HBV surface antigen
APC: Age-Period-Cohort model
ASRs: Age-standardized rates
*C. sinensis*: *Clonorchis sinensis*
CC: Cholangiocarcinoma
cHCC-CC: Combined Hepatocellular and cholangiocarcinoma
DCN: Death Certification Notifications
DF: Degree of freedom
EAPC: Estimated annual percent change
EAPCs: Estimate annual percent changes
HBs-Ag: HBV surface antigens
HBV: Hepatitis B virus
HCC: Hepatocellular carcinoma
HCV: Hepatitis C virus
ICD-10: International Classification of Diseases-10th revision
M:I: The Ratio of Mortality to Incidence
PLC: Primary Liver Cancer
RR: Relative Risk
Δ Deviance: Increase in deviance from the APC model
Δ DF: Increase in DF from the APC model
